# Identification, pyramid and candidate genes of QTLs for associated traits based on a dense erect panicle rice CSSL-Z749 and five SSSLs, three DSSLs and one TSSL

**DOI:** 10.1186/s12284-021-00496-7

**Published:** 2021-06-16

**Authors:** Dachuan Wang, Kai Zhou, Siqian Xiang, Qiuli Zhang, Ruxiang Li, Miaomiao Li, Peixuan Liang, Naz Farkhanda, Guanghua He, Yinghua Ling, Fangming Zhao

**Affiliations:** Rice Research Institute, Academy of Agricultural Sciences, Southwest University, 400715 Chongqing, PR China

**Keywords:** Rice, Chromosome segment substitution lines, Panicle related traits, QTL, Fine mapping, Additive and epistatic effects

## Abstract

**Background:**

Seed-set density is an important agronomic trait in rice. However, its genetic mechanism is complex. Chromosome segment substitution lines (CSSLs) are ideal materials for studying complex traits.

**Results:**

A rice CSSL, Z749, with a dense and erect panicle phenotype, was identified among progeny of the recipient parent Nipponbare and the donor parent Xihui 18. Z749 carried seven substitution segments (average length 2.12 Mb). Compared with Nipponbare, Z749 showed significant increases in the numbers of primary (NPB) and secondary branches (NSB), number of spikelets (SPP) and grains per panicle (GPP), seed-set density (SSD), and decrease in panicle length (PL). A secondary F_2_ population derived from a cross between Nipponbare and Z749 was used to map quantitative trait loci (QTLs) for associated traits. Fifteen QTLs distributed on chromosomes 5, 7, 8, and 10 were detected. The QTL *qPL7* might be an allele of *OsFAD8* and the remaining 14 QTLs (e.g., *qSSD5* and *qSSD10* etc.) might be novel. Fourteen QTLs were verified using five single-segment substitution lines (SSSLs). The seed-set density of Z749 was controlled predominantly by one major QTL (*qSSD10*) and two minor QTLs (*qSSD5* and *qSSD8*). The QTLs *qSSD10*, *qSSD5*, and *qSSD8* were fine-mapped to intervals of 1.05, 1.46, and 1.53 Mb on chromosomes 10, 5, and 8, respectively. Analysis of QTL additive effects indicated that *qSSD5*, *qSSD8*, and *qSSD10* from Xihui18 increased seed-set density of Z749 by 14.10, 11.38, and 5.11 spikelets per 10 cm panicle, respectively. Analysis of QTL epistatic effects revealed that pyramiding of *qSSD5* and *qSSD8*, *qSSD5* and *qSSD10*, *qSSD8* and *qSSD10*, and *qSSD5*, *qSSD8* and *qSSD10* produced novel genotypes with increased seed-set density.

**Conclusions:**

Inheritance of seed-set density in Z749 was controlled predominantly by one major QTL (*qSSD10*) and two minor QTLs (*qSSD5* and *qSSD8*). Then, they were fine-mapped to intervals of 1.05, 1.46, and 1.53 Mb on chromosomes 10, 5, 8, respectively. Two MAPK genes (*OsMPK9* and *OsMPK17*) and one gene (candidate gene 6) involved in auxin metabolism might be candidate genes for *qSSD5*, and *OsSAUR32* might be the candidate gene for *qSSD8.* Pyramiding of *qSSD5*, *qSSD8*, and *qSSD10* enhanced seed-set density.

**Supplementary Information:**

The online version contains supplementary material available at 10.1186/s12284-021-00496-7.

## Background

Rice (*Oryza sativa* L.) is an important cereal crop and a staple food for more than 50 % of the global population (Gathala et al. 2011). Improvement of the yield continues to be a crucial focus in rice breeding. Panicle architecture is an important agronomic trait that affects seed-set density and is strongly associated with grain yield. Assuming no changes to other components, an increase in seed-set density can improve rice yield. However, the genetic mechanism for seed-set density is complex and controlled by multiple genes.

In recent decades, a number of genes have been shown to affect the architecture of the rice panicle. Some of these genes are involved in the hormone signaling pathways of phytohormones, such as cytokinins, auxin, and brassinolactone. Cytokinins regulate the size of reproductive meristematic tissue, and auxin is involved in the formation of meristematic tissue, thereby affecting panicle development (Wang et al. 2018). Genes associated with the cytokinin metabolism pathway include *Gn1a* (*GRAIN NUMBER 1 a*; *OsCKX2*), *DST* (*DROUGHT AND SALT TOLERANCE*), *SMG1* (*SMALL GRAIN 1*), *GSN1* (*GRAIN SIZE AND NUMBER 1*), *OsER1* (*ERECTA 1*), *DEP1* (*DENSE AND ERECT PANICLE 1*) and *LP* (*LARGER PANICLE*). The gene *Gn1* *a* encodes an enzyme that catalyzes the degradation of active cytokinins. Reduced *Gn1a* expression may lead to accumulation of cytokinins in the inflorescence meristems, thereby increasing the number of spikelets and leading ultimately to denser panicles (Ashikari et al. 2005). The genes *DST*, *SMG1*, *GSN1*, *OsER1*, *DEP1*, and *LP* can affect the cytokinin content in reproductive meristems by direct or indirect upregulating of the transcription of *Gn1a*, and can therefore affect panicle development (Duan et al. 2014; Guo et al. 2018, 2020; Huang et al. 2009; Li et al. 2011, 2013; Liu et al. 2018; Piao et al. 2009; Zhang and Yuan 2014). Genes associated with the auxin metabolism pathway include *PAY1* (*PLANT ARCHITECTURE AND YIELD 1*), *LAX1* (*LAX PANICLE 1*), *SPA* (*SMALL PANICLE*), and *ASP1* (*ABERRANT SPIKELET AND PANICLE1*). *PAY1* may improve the architecture of the plant panicle by altering distribution of endogenous indole-3-acetic acid and affecting polar auxin transport activity (Zhao et al. 2015). *LAX1*, *SPA*, and *ASP1* are indicated to play crucial roles in auxin-mediated panicle development but their mode of action is not clear (Keishi et al. 2003; Shen et al. 2010; Yoshida et al. 2012; Zhang and Yuan 2014). *DWARF 11* (*D11*, *CPB1*) is involved in the brassinolide biosynthetic pathway. The *cpb1* mutant shows phenotypes of increased BR-sensitivity, clustered primary branches of the panicle, and smaller seeds (Wu et al. 2016). However, it remains unclear how brassinolide affects panicle development. In addition, certain genes regulate rice panicle development through other pathways, such as *DEP2*, *DEP3*, *OsMFT1* (*MOTHER OF FT AND TFL 1*), and *FUWA *(Chen et al. 2015; Li et al. 2010; Liu et al. 2015; Qiao et al. 2011; Song et al. 2018). Although many genes associated with panicle architecture have been cloned, they are insufficient to explain the complexity of the genetic mechanism. Therefore, it is necessary to identify additional genes that influence the dense panicle architecture of rice.

Chromosome segment substitution lines (CSSLs) are ideal materials to use for genetic research of complex traits and genetic resources for crop improvement (Balakrishnan et al. 2019; Zhang et al. 2019). Here, we identified a rice CSSL, Z749, with a dense and erect panicle phenotype, derived from ‘Nipponbare’ as the recipient parent and ‘Xihui 18’ as the donor parent. We characterized Z749 genetically and mapped quantitative trait loci (QTLs) for associated traits using a secondary F_2_ population derived from a cross between Nipponbare and Z749. In addition, we developed single-segment (SSSLs), double-segment (DSSLs), and triple-segment substitution lines (TSSLs) for each QTL in the F_3_ generation using marker-assisted selection (MAS). We verified the accuracy of QTL mapping using SSSLs. We conducted analysis of the additive and epistatic effects of QTLs on seed-set density and assessed the effect of QTL pyramiding using DSSLs and TSSLs. In addition, the QTLs *qSSD5*, *qSSD8*, and *qSSD10* were fine-mapped and candidate genes were analysed.

## Results

### Identification of substitution segments in Z749

**Fig. 1:**
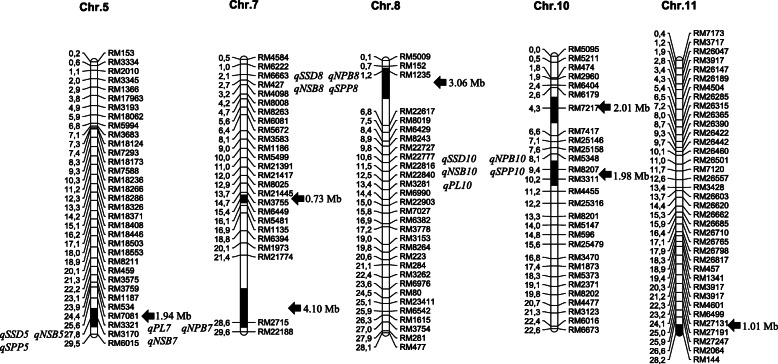
Substitution segments and detected QTLs in Z749. Physical distances (Mb) and mapped QTLs are marked at the left of each chromosome; markers and substitution length (black arrow direction) are displayed to the right of each chromosome. PL: panicle length, NPB: number of primary branches, NSB: number of secondary branches, SPP: spikelet number per panicle, SSD: seed-set density

Identification of the substitution segments and detection of the genetic background purity were performed on 10 individuals of the Z749 lines using all simple sequence repeat (SSR) markers located on the seven substitution segments of Z749 and 24 SSR markers, which were in turn located outside the substitution segments. The results showed that Z749 was homozygous and no additional residual segment was detected. Seven chromosomal substitution segments derived from Xihui 18 were located on chromosomes 5, 7, 8, 10, and 11. Chromosomes 7 and 10 each contained two substitution segments. The total substitution length was 14.83 Mb, the longest substitution was 4.10 Mb, the shortest substitution was 0.73 Mb, and the mean substitution length was 2.12 Mb (Fig. 1).

### Phenotypes of CSSL-Z749

**Fig. 2:**
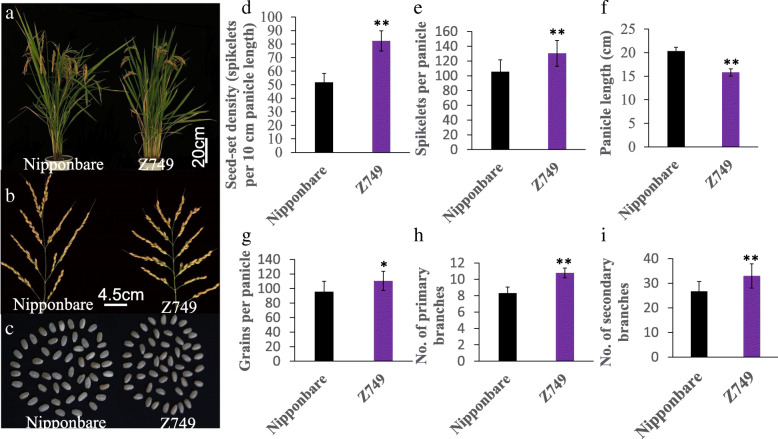
Phenotype of Nipponbare and Z749. **a** Plant type of Nipponbare and Z749. **b** Panicle of Nipponbare and Z749. **c** Brown rice of Nipponbare and Z749. **d** Seed-set density. **e** Spikelet number per panicle. **f** Panicle length. **g** Grain number per panicle. **h** Number of primary branches. **i** Number of secondary branches

Compared with Nipponbare, CSSL-Z749 showed significant increase in number of grains per panicle, number of spikelets per panicle, number of primary branches, number of secondary branches, and seed-set density (Fig. 2A, B), which were increased by 15.51 %, 23.92 %, 29.57 %, 23.65 %, and 59.42 % (Fig. 2G, E, H, I, D), respectively. The seed-set density of Z749 was 82.34 spikelets per 10 cm panicle length, whereas that of Nipponbare was 51.65 (Fig. 2D). The panicle length of Z749 was decreased significantly compared with that of Nipponbare (Fig. 2B, F). Therefore, the increased seed-set density of Z749 was caused predominantly by a reduction in the panicle length and increases in the numbers of branches and spikelets per panicle. For other agronomic traits, such as heading date and 1000-grain weight (Fig. 2A, C), no significant differences were observed between Z749 and Nipponbare.

### QTL mapping for seed-set density related traits in secondary F_2_ population of Nipponbare/Z749

The substitution segments of Z749 carried 15 QTLs for traits associated with seed-set density. The percentage phenotypic variation explained by individual QTLs ranged from 4.68 to 21.01 %. Seven QTLs were major and contributed to panicle length (*qPL7* and *qPL10*), number of primary branches (*qNPB7*), number of secondary branches (*qNSB7* and *qNSB10*), number of spikelets per panicle (*qSPP10*), and seed-set density (*qSSD10)*. The remaining eight QTLs showed minor effects (Table 1; Fig. 1).

**Table 1:** QTL for rice seed-set density related traits detected in Z749

Trait	QTL	Chr.	Linked marker	Additive effect	Var. (%)	*P*-value	Possible alleles
Panicle length (cm)	*qPL7*	7	RM2715	1.47	13.98	0.0020	*OsFAD8* (Nair et al. 2009)
	*qPL10*	10	RM8207	-2.09	21.01	0.0192	-
Number of primary branch	*qNPB7*	7	RM2715	0.65	13.54	0.0032	-
	*qNPB8*	8	RM1235	0.59	5.69	0.0018	*OsREL2* (Kwon et al. 2012)
	*qNPB10*	10	RM8207	0.56	9.16	0.0103	-
Number of secondary branch	*qNSB5*	5	RM3170	1.79	5.46	0.0157	-
	*qNSB7*	7	RM2715	3.13	13.48	0.0038	-
	*qNSB8*	8	RM1235	3.26	7.38	0.0081	*OsREL2* (Kwon et al. 2012)
	*qNSB10*	10	RM8207	2.93	12.07	0.0059	-
Spikelet number per panicle	*qSPP5*	5	RM3170	7.24	5.67	0.0136	*OsPUP7* (Qi and Xiong 2013)
	*qSPP8*	8	RM1235	10.30	4.68	0.0278	*-*
	*qSPP10*	10	RM8207	12.07	13.01	0.0031	-
Seed-set density	*qSSD5*	5	RM3170	3.30	6.15	0.0104	*OsPUP7* (Qi and Xiong 2013)
(Spikelets per 10 cm panicle length)	*qSSD8*	8	RM1235	5.68	7.44	0.0081	*-*
	*qSSD10*	10	RM8207	6.88	20.20	0.0002	-

The panicle length of Z749 was controlled by one major QTL (*qPL7*) that increased panicle length by 1.47 cm and one major QTL (*qPL10*) that reduced panicle length by 2.09 cm. The number of spikelets per panicle of Z749 was controlled by one major QTL (*qSPP10*) and two minor QTLs (*qSPP5* and *qSPP8*) that increased the trait. The seed-set density of Z749 was controlled by one major QTL (*qSSD10*) and two minor QTLs (*qSSD5* and *qSSD8*) that increased the trait.

In order to decide which QTLs should be studied in depth,we primarily wanted to define which genes for the according traits have been cloned previously at all the substitution segments. Finally, five QTLs revealed to contain reported genes (Kwon et al. 2012; Nair et al. 2009; Qi and Xiong 2013) (Table 1).

**Fig. 3:**
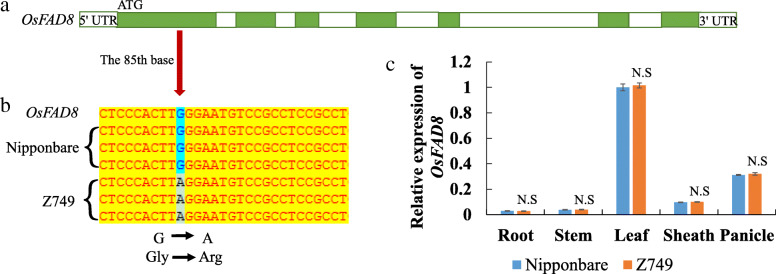
Blast analysis and expression level of *OsFAD8* in Nipponbare and Z749. **a** Sketch map of *OsFAD8* (*Os07g0693800*), **b** difference in sequences of *OsFAD8* between Nipponbare and Z749. Green boxes indicate exons, white boxes indicate introns and 3’ untranslated region; red arrows indicate the alteration of a codon. **c** The relative expression levels of *OsFAD8* in root, stem, leaf, sheath and panicle between Nipponbare and Z749

With regard to the possible alleles *OsFAD8* (*FATTY ACID DESATURASE*; *Os07g0693800*), *OsREL2* (*ROLLED AND ERECT LEAF 2*; *Os08g0162100*), and *OsPUP7* (*PURINE PERMEASE 7*; *Os05g0556800*) in Table 1, we performed comparative DNA sequencing analysis and qRT-PCR analysis between Nipponbare and Z749. However, for *OsREL2* and *OsPUP7*, no differences were observed in both the DNA sequences and expression levels between Nipponbare and Z749 (Figure S1A, B), which indicates that these were not the candidate genes for *qNPB8*, *qNSB8*, *qSPP5*, and *qSSD5*. For *OsFAD8*, a single nucleotide polymorphism (SNP) difference in the DNA sequence between Nipponbare and Z749 was detected. The 85th base (the 85th base of the first exon) was mutated from G in Nipponbare to A in Z749. This transition caused a change of amino acid from Glycine to Arginine (Fig. 3A,B). However, its expression level was not different between Nipponbare and Z749 (Fig. 3C), Thus, *qPL7* might be an allele of *OsFAD8*.

### Verification of QTLs using SSSLs and analysis of epistatic effects of QTLs for seed-set density using DSSLs and TSSL

**Fig. 4:**
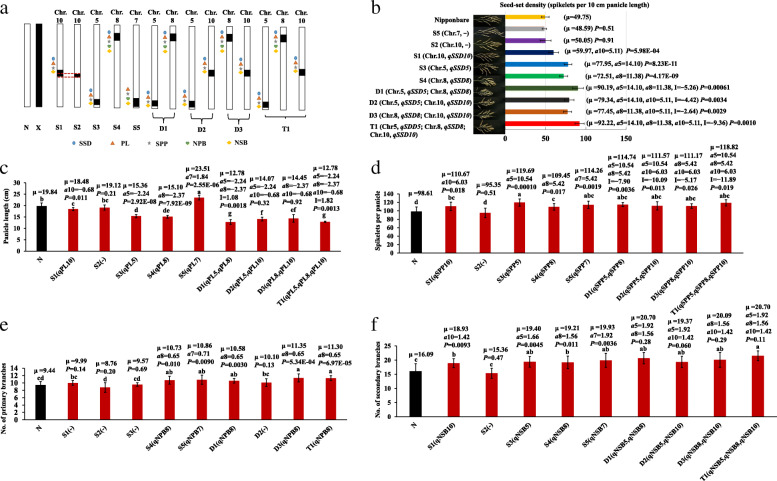
Analysis of additive and epistatic effects of QTLs for seed-set density and associated traits in S1-S5, D1-D3 and T1. **a** Sketch map of developed SSSLs (S1-S5), DSSLs (D1-D3) and TSSL (T1). N denotes recipient Nipponbare; X denotes donor Xihui18; S: SSSL, D: DSSL, T: TSSL. **b** Seed-set density (SSD); **c** panicle length (PL); **d** Spikelets per panicle (SPP); **e** Number of primary branches (NPB); **f** Number of secondary branches (NSB). Different lower-case letters indicate a significant difference (*P* < 0.05) as determined by Duncan’s multiple comparisons. µ is the mean value, a_i_ denotes the additive effect of QTLs, I denotes the additive × additive epistatic effect between QTLs. The *P*-value for a SSSL indicates the probability of a significant difference between the SSSL and Nipponbare, and the SSSL carried a QTL (Student’s t-test, *p* < 0.05). The *P*-value for a DSSL and TSSL indicates the probability of an epitatic effect between QTLs in the DSSL or TSSL, i.e., (Nipponbare + DSSL_ij_) and (SSSL_i_ and SSSL_j_), and (Nipponbare + Nipponbare + TSSL_ijk_) and (SSSL_i_ and SSSL_j_ + SSSL_k_) (Student’s t-test, *p* < 0.05). S1:RM5348–RM8207-RM3311–RM4455 (Chr.10); S2: RM8207–RM3311–RM4455 (Chr.10); S3: RM3321–RM3170–RM6015 (Chr.5); S4: RM152–RM1235–RM22617 (Chr.8); S5: RM21774–RM2715–RM22188 (Chr.7). D1: RM3321–RM3170–RM6015 (Chr.5), RM152–RM1235–RM22617 (Chr.8); D2: RM3321–RM3170–RM6015 (Chr.5), RM5348–RM8207-RM3311–RM4455 (Chr.10); D3: RM152–RM1235–RM22617 (Chr.8), RM5348–RM8207-RM3311–RM4455 (Chr.10); T1: RM3321–RM3170–RM6015 (Chr.5), RM152–RM1235–RM22617 (Chr.8), RM5348–RM8207-RM3311–RM4455 (Chr.10)

On the basis of the QTL mapping, we developed five SSSLs (S1, S2, S3, S4, and S5), three DSSLs (D1, D2, and D3), and one TSSL (T1) in the F_3_ population by MAS (Fig. 4A). The substitution segments of S1 and S2 were located on chromosome 10, with overlapping substitution segments, but the length of the substitution segment of S2 was shorter than that of S1 (Fig. 4A). Four QTLs were validated by S1, namely *qSSD10*, *qPL10*, *qSPP10*, and *qNSB10* (Fig. 4B-D, F), whereas one minor QTL (*qNPB1*0) (Fig. 4E; Table 1) was not detected by S1. These QTLs were not detected by S2 (Fig. 4B-F), which indicates that the QTLs were located in the substitution interval of RM5348–RM8207–RM3311 (Fig. 4A). The substitution segment of S3 was located on chromosome 5 on which four QTLs (*qSSD5*, *qPL5*, *qSPP5*, and *qNSB5*) were detected (Fig. 4B-D, F). The S4 substitution segment was located on chromosome 8 on which five QTLs were identified, namely *qSSD8*, *qPL8*, *qSPP8*, *qNPB8*, and *qNSB8* (Fig. 4B-F). The substitution segment of S5 was located on chromosome 7 and four QTLs were identified, which consisted of *qPL7*, *qSPP7*, *qNPB7*, and *qNSB7* (Fig. 4C-F). The QTLs *qPL5*, *qPL8*, and *qSPP7* were detected in the corresponding SSSLs S3, S4, and S5, respectively (Fig. 4C, D), while not detected in the secondary F_2_ segregation population of Nipponbare/Z749 (Table 1). The result suggests that the SSSL showed a higher QTL detection efficiency. Thus, 14 of the 15 QTLs identified in 2018 were verified by SSSLs in 2020, indicating that the QTLs were mapped accurately.

The DSSL D1 carried substitution segments of chromosomes 5 and 8. The substitution segment of chromosome 5 contained *qSSD5*, the additive effect of which was 14.10 spikelets per 10 cm panicle length. The substitution fragment of chromosome 8 contained *qSSD8*, whose additive effect was 11.38 spikelets per 10 cm panicle length. The epistatic effect between *qSSD5* and *qSSD8* was − 5.26. Thus, the pyramiding of *qSSD5* and *qSSD8* produced a genetic effect of 20.22 spikelets per 10 cm panicle length and therefore yielded significant denser panicles than those of S3 and S4 (Fig. 4B). The DSSL D2 carried substitution segments of chromosomes 5 and 10. The epistatic effect between *qSSD5* (*a* = 14.10) and *qSSD10* (*a* = 5.11) was − 4.42. Thus, pyramiding of *qSSD5* and *qSSD10* produced a genetic effect of 14.79 spikelets per 10 cm panicle length in D2, which was larger than the additive effect of S3 (containing *qSSD5*) and S1 (containing *qSSD10*) (Fig. 4B). The DSSL D3 carried substitution segments of chromosomes 8 and 10. Pyramiding of *qSSD8* (*a* = 11.38) and *qSSD10* (*a* = 5.11) produced an epistatic effect of − 2.64, which resulted in an increase of 13.85 spikelets per 10 cm panicle length in D3. Thus, pyramiding of *qSSD8* and *qSSD10* also yield denser panicles than S4 (containing *qSSD8*) and S1 (containing *qSSD10*) (Fig. 4B). The TSSL T1 carried substitution segments of chromosomes 5, 8, and 10. Pyramiding of *qSSD5*, *qSSD8*, and *qSSD10* produced an epistatic effect of − 9.36. The genetic effect of the three QTLs in T1 for seed-set density was 21.23 spikelets per 10 cm panicle length. Thus, pyramiding of *qSSD5*, *qSSD8*, and *qSSD10* also yielded significant denser panicles than S3, S4, and S1. Taken together, all combinations of *qSSD5* and *qSSD8*, *qSSD5* and *qSSD10*, *qSSD8* and *qSSD10*, and *qSSD5*, *qSSD8* and *qSSD10* resulted in increase in seed-set density (Fig. 4B).

The number of secondary branches and spikelets of per panicle of S1, S3, S4, D1, D2, D3, and T1 was significantly higher than that of Nipponbare (Fig. 4F, D). The number of primary branches of S4, D1, D3, and T1 was significantly higher than that of Nipponbare (Fig. 4E). The panicle length of S1, S3, S4, D1, D2, D3, and T1 was significantly lower than that of Nipponbare (Fig. 4C). Thus, the seed-set densities of S1, S3, S4, D1, D2, D3, and T1 were significantly higher than that of Nipponbare (Fig. 4B). In accordance with this finding, *qNPB8*, *qNSB10*, *qNSB8*, *qNSB5*, *qSPP10*, *qSPP5*, and *qSPP8* all showed additive effects to increase the trait values, whereas *qPL10*, *qPL5*, and *qPL8* showed an additive effect to reduce panicle length. The QTLs *qSSD5*, *qSSD8*, and *qSSD10* showed additive effects that increased seed-set density.

### Genetic analysis of *qSSD5*, *qSSD8*, and *qSSD10*

For genetic analysis and fine mapping of *qSSD5*, *qSSD8*, and *qSSD10*, F_3_ populations were constructed by respective recombinant plants. For *qSSD5*, the chi-square test showed that the numbers of low seed-set density (117) and high seed-set density (383) individuals corresponded to a separation ratio of 1:3 (χ^2^ = 0.60 < χ^2^_(0.05,1)_ = 3.84); for *qSSD8*, the chi-square test showed that the numbers of low seed-set density (37) and high seed-set density (143) individuals corresponded to the same separation ratio of 1:3 (χ^2^ = 2.86 < χ^2^_(0.05,1)_ = 3.84); for *qSSD10*, the chi-square test showed that the numbers of high seed-set density (92) and low seed-set density (308) individuals corresponded to the same separation ratio of 1:3 (χ^2^ = 0.75 < χ^2^_(0.05,1)_ = 3.84). These results suggest that *qSSD5* and *qSSD8* showed dominant gene action, whereas *qSSD10* showed recessive gene action.

### Fine mapping of *qSSD5* and analysis of DNA sequence and qRT-PCR of candidate genes

**Fig. 5:**
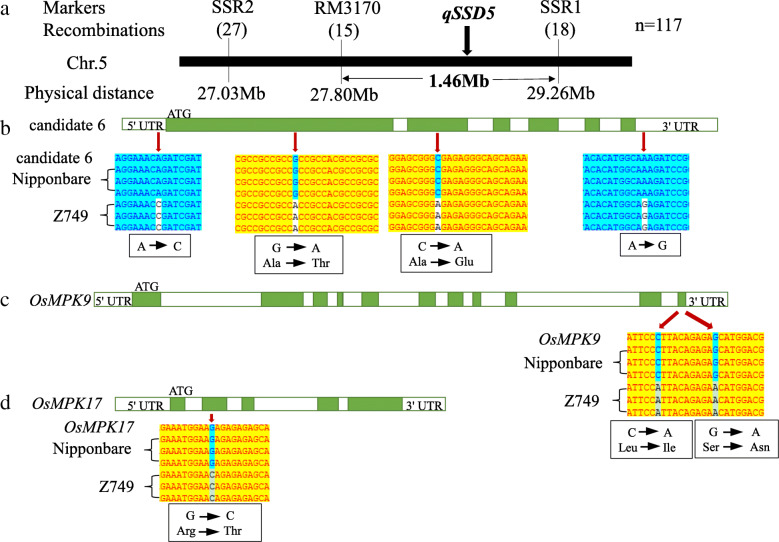
Fine mapping of *qSSD5* and analysis of candidate genes 5, 6 and 7. **a** Fine mapping of *qSSD5*. **b**–**d** DNA sequences of candidate gene 6, candidate gene 5 (*OsMPK9*), and candidate gene 7 (*OsMPK17*) in Z749 compared with those of Nipponbare. Green indicates the coding sequence (CDS) of exons and white indicates the introns and untranslated regions (UTR) of the genes. The red arrow indicates the site of a SNP

**Fig. 6:**
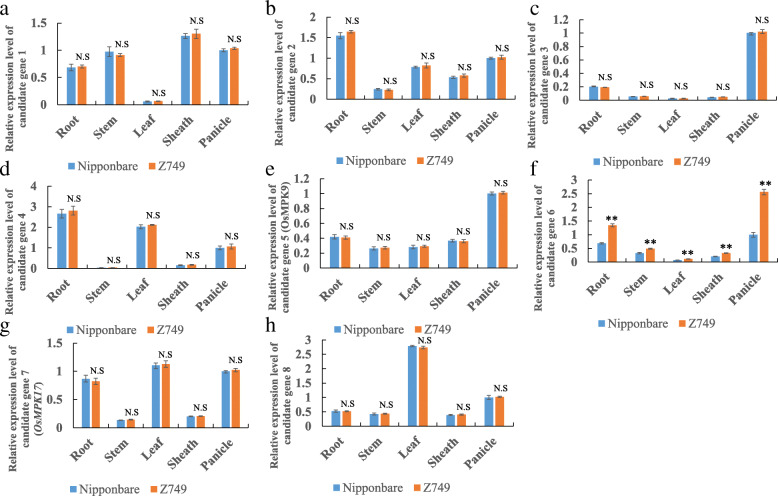
Relative expression level of eight candidate genes for *qSSD5* between Nipponbare and Z749. **a**-**h** Relative expression level of candidate genes 1–8 in root, stem, leaf, sheath and panicle between Nipponbare and Z749

Two newly developed SSR markers and 117 recessive individuals with low seed-set density from the F_3_ population were used for fine mapping of *qSSD5*. The QTL *qSSD5* was delimited within a 1.46 Mb interval between the molecular markers RM3170 and SSR1 on chromosome 5 (Fig. 5A). Considering that the cloned genes related to rice panicle development mainly involved in plant hormone signaling pathways or MAPK cascades (Guo et al. 2018; Zhang and Yuan 2014), we checked all the genes in the 1.46 Mb region of *qSSD5*, and found that 8 genes are related to plant hormones and MAPK pathway. The other genes include hypothetical proteins, unknown functional proteins, non-protein coding transcript and Cyclin-like F-box domain containing protein and etc. Therefore, we preliminarily selected these 8 genes as candidate genes. Furthermore, by DNA sequencing of these genes including 3000 bp before the start codon ATG (promoter regions) and 1500 bp after the stop codon between Nipponbare and Z749, candidate gene 1 (auxin-responsive protein), candidate gene 2 (*OsIAA19*), candidate gene 3 (gibberellin 2-beta-dioxygenase), candidate gene 4 (auxin response factor 15), and candidate gene 8 (OsGH3.5; Probable indole-3-acetic acid-amido synthetase) showed no difference between the two lines. Besides, qRT-PCR results showed that the expression levels of these 5 genes in panicle, root, stem, leaf and sheath were all no significant difference between Nipponbare and Z749 (Fig. 6A, B, C, D, H). These results showed that these five genes were not candidate genes for *qSSD5*. The DNA sequence of candidate gene 5 (*OsMPK9*), candidate gene 6 (auxin efflux carrier component), and candidate gene 7 (*OsMPK17*) differed between Nipponbare and Z749. For candidate gene 6, four SNP differences were detected, of which two were located in the coding sequence (CDS) and caused changes in amino acid, and one each was located in the 5′ untranslated region (UTR) and the 3′ UTR (Fig. 5B). Especially, the expression levels of the candidate gene 6 were significantly higher in panicle, root, stem, leaf and sheath in Z749 than in Nipponbare (Fig. 6F). For candidate gene 5 (*OsMPK9*), two SNP differences were detected in the CDS sequence between Nipponbare and Z749 and both caused amino acid changes (Fig. 5C). For candidate gene 7 (*OsMPK17*), one SNP difference in the CDS sequence was observed between Nipponbare and Z749 and caused an amino acid change from Arginine in Nipponbare to Threonine in Z749 (Fig. 5D). However, the expression levels of the two genes in panicle, leaf, root, stem and sheath displayed no significant differences between Nipponbare and Z749 (Fig. 6E, G). Thus, candidate gene 5 (*OsMPK5*), candidate gene 6, and candidate gene 7 (*OsMPK17*), were potential candidate genes for *qSSD5*. In particular, the candidate gene 6 should be the best one for *qSSD5.*

### Fine mapping of *qSSD8* and sequence analysis and qRT-PCR of candidate genes

**Fig. 7:**
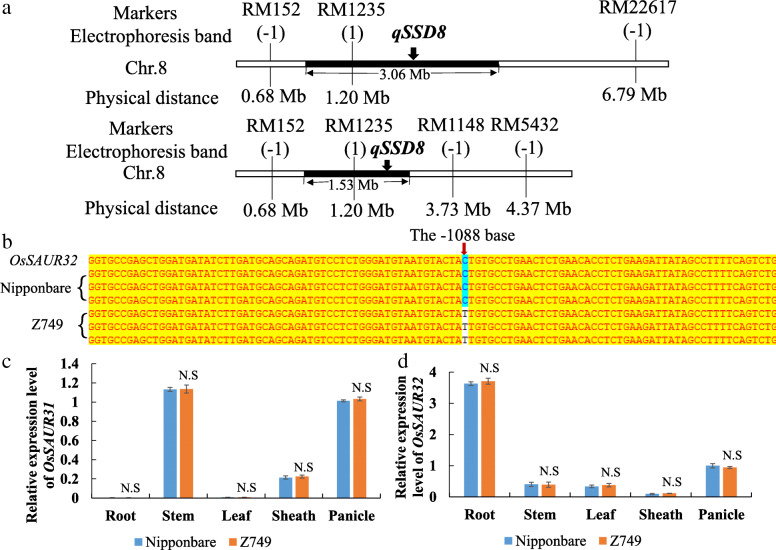
Fine mapping of *qSSD8* and analysis of DNA sequence and expression levels of its candidate genes. **a** Fining mapping of *qSSD8*. **b** Nucleotide sequence of *OsSAUR32* (*LOC_Os08g02530*) in Z749 compared with that of Nipponbare. −1 indicates that the band for S4 was identical to that of Nipponbare; 1 indicates that the band for S4 was identical with Xihui18. **c**, **d** The relative expression levels of *OsSAUR31* and *OsSAUR32* in root, stem, leaf, sheath and panicle between Nipponbare and Z749, respectively

Twenty pairs of SSR markers were newly developed in the substitution segment of *qSSD8*, of which two polymorphic markers were detected between Nipponbare and Z749. However, the electrophoresis bands for RM1148 and RM5432 were identical with those of Nipponbare. Thus, the estimated length and maximum length of the substitution segment of *qSSD8* were further shortened from the original 3.06 Mb and 6.11 Mb to 1.53 Mb and 3.05 Mb, respectively (Fig. 7A). By candidate gene prediction in the substitution interval, the small auxin-up RNAs *OsSAUR31* (*LOC_Os08g02520*) and *OsSAUR32* (*LOC_Os08g02530*) were identified as candidate genes for *qSSD8*. Further sequencing revealed that only *OsSAUR32* differed between Nipponbare and Z749. The − 1088th base (located in the promoter region) was mutated from C in Nipponbare to T in Z749 (Fig. 7B). Furthermore, qRT-PCR analysis showed that the expression levels of *OsSAUR31* and *OsSAUR32* exhibited no differences in panicle, root, stem, leaf and sheath between Nipponbare and Z749 (Fig. 7C, D). Thus, *OsSAUR32* might be a putative candidate gene for *qSSD8*.

### Overlapping substitution mapping of *qSSD10*

**Fig. 8:**
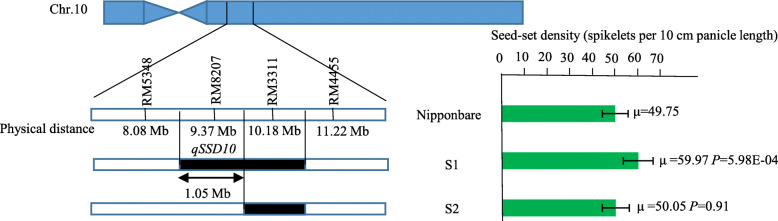
Fine-mapping of *qSSD10*. Black regions indicate the estimated length of substitution segment. S: SSSL. µ is the mean value. *P* is the probability value from a Student’s t-test for the difference in seed-set density between Nipponbare and S1 and S2

Given that too few polymorphic markers were detected, fine mapping of *qSSD10* was conducted by using overlapping substitution segments of S1 and S2. The substitution segment of S2 was shorter than that of S1. The SSSL S1 carried *qSSD10* and showed a seed-set density of 59.97 spikelets per 10 cm panicle length, which differed significantly from that of Nipponbare (49.75 spikelets per 10 cm panicle length). In contrast, S2 did not harbor *qSSD10* and showed a seed-set density of 50.05 spikelets per 10 cm panicle length, which did not differ significantly from that of Nipponbare (Fig. 8). Therefore, *qSSD10* was subject to further fine-mapping within the substitution interval of RM5348–RM8207–RM3311, with an estimated length of 1.05 Mb and maximum length of 2.10 Mb (Fig. 8).

## Discussion

### The Z749 is a useful genetic resource for rice breeding

To a certain extent, a higher seed-set density is beneficial to increase the grain number and improve grain filling, thereby increasing rice yield. However, seed-set density is a complex trait controlled by multiple genes, and is affected by panicle length, number of primary branches, number of secondary branches, and number of spikelets per panicle. Rice CSSLs represent a chromosome segment introgression line library composed of distant genotypes in a superior genetic background, and provide a valuable genetic resource for research into theoretical aspects and the application of complex traits (Furuta et al. 2014). Recently, Li et al. (2019) developed a CSSL population consisting of 75 lines from a cross between XQZB and ZH9308 to identify the QTLs that affect yield-related traits, such as 1000-grain weight, grain length, and grain width. We identified a rice CSSL, Z749, with dense and erect panicles, which carried seven substitution segments from the donor parent Xihui 18 based on the recipient parent Nipponbare. The increased seed-set density of Z749 was caused predominantly by a reduction in panicle length and increases in the numbers of primary and secondary branches, and the number of spikelets per panicle. Z749 harbored many favorable traits useful for rice breeding, in addition to a high seed-set density, including an increased number of spikelets per panicle. Furthermore, no differences in 1000-grain weight or numbers of panicles per plant were observed between Nipponbare and Z749. Therefore, Z749 is a potentially valuable genetic resource for rice breeding.

### QTL identification and dissection of complex traits with the Z749, and comparison with previously reported genes

Given that Z749 carried seven substitution segments from the donor parent Xihui 18, it was essential to determine the genes distributed in the seven substitution segments and to decompose these segments into different SSSLs. Therefore, we constructed a secondary F_2_ population by crossing Nipponbare and Z749 to map QTLs for associated traits. We determined that 15 QTLs were distributed on four substitution segments of Z749. The seed-set density of Z749 was controlled by one major QTL (*qSSD10*) and two minor QTLs (*qSSD5* and *qSSD8*). Compared with the results of previous research, *OsPUP7* (27.690–27.691 Mb) was located in the mapping region of *qSPP5* and *qSSD5*. The distance of the linkage marker RM3170 (27.80 Mb) of *qSPP5* and *qSSD5* from *OsPUP7* was 0.11 Mb. *OsPUP7* encodes a permease with associated purine transport activity involved in cytokinin transport. The *pup7* mutant shows increased plant height, reduced number of spikelets per panicle, and delayed flowering (Qi and Xiong 2013). However, no difference in both DNA sequence and gene expression level (Figure S1B) for *OsPUP7* was detected between Z749 and Nipponbare, which indicates that *OsPUP7* was not the candidate gene for *qSPP5* and *qSSD5*. The QTLs *qNPB8* and *qNSB8* were linked with RM1235 (1.20 Mb), and increased the number of primary branches by 0.59 and the number of secondary branches by 3.26. The gene *OsREL2* (3.667–3.675 Mb) was located in this interval, just 2.47 Mb from RM1235. The *osrel2* mutant displays a higher number of primary branches but fewer secondary branches compared with those of the wild type (Kwon et al. 2012). However, no difference in the sequence and gene expression level (Figure S1A) of *OsREL2* between Z749 and Nipponbare was detected, which indicates that *qNPB8* and *qNSB8* were not alleles of *OsREL2.* The QTL *qPL7* might be an allele of *OsFAD8* (29.531–29.534 Mb), given the 0.94 Mb distance of *OsFAD8* from RM2715 (28.59 Mb). *OsFAD8* encodes a ω-3 fatty acid desaturases. The *osfad8* mutant exhibits decreased plant and panicle length (Nair et al. 2009). Sequencing revealed a difference in the 85th base of the first exon of *OsFAD8* between Nipponbare and Z749, which caused a change in amino acid from Glycine in Nipponbare to Arginine in Z479. And, the gene expression level of *OsFAD8* was not different in root, stem, leaf, sheath and panicle between Nipponbare and Z749. The other identified QTLs (e.g., *qSSD10*) have not been studied in any detail. Whether the QTLs correspond to the aforementioned alleles requires verification by further genetic complementation experiments. With regard to whether these QTLs might be cloned alleles, we developed corresponding SSSLs and observed that each line carried many favorable traits useful for breeding (Fig. 4). Consequently, compared with the aforementioned mutants, which often carry adverse traits, the SSSLs can be utilized readily in molecular breeding. As for the non-cloned QTLs as *qSPP5*, *qSSD5*, *qSSD10*, and *qSPP10*, they can be further fine-mapped and cloned, thereby allowing the underlying molecular mechanism to be investigated in more detail.

### SSSLs, DSSLs, and TSSLs are suitable materials for target QTLs analysis

Single-segment substitution lines are ideal materials for genetic analysis and breeding because they differ in only one substitution segment compared with the recipient parent. Moreover, SSSLs are homozygous and can be used as permanent populations (Zhao et al. 2007). From the results of the primary QTL mapping, we developed five SSSLs (S1 to S5) using a MAS method. These lines were used to validate the stability of 14 QTLs, for which the repeatability of detection was 93.33 %. In addition, *qNPB7* was also detected by Wang et al. (2020). Thus, these QTLs were genetically stable. In S5 we detected a minor QTL (*qSPP7*), in S3 one QTL (*qPL5*), and in S4 one QTL (*qPL8*), but these QTLs were not detected in the secondary F_2_ segregation population of Nipponbare/Z749 in 2018. These results reveal that the SSSL shows a higher sensitivity of QTL detection, a finding confirmed previously by Zhao et al. (2016b) and Eshed and Zamir (1995). However, one QTL (*qNPB10*) was not detected repeatedly, which suggests that the detection of some minor QTLs depends on the environment. Liu et al. (2008) highlighted that for a specific environment, the total effect of a QTL includes the main effect and the QTL × environment (Q×E) interaction effect for that environment. In this manner, *qNPB10* might only show a Q×E interaction effect. Zhao et al. (2016b) observed that different agronomic traits displayed different Q×E interactions and that the Q×E interaction effect is specific to a particular environment. Thus, different SSSLs that differ in the QTLs carried should be treated differently in different applications. Certain SSSLs that carry stable favourable QTLs will be more valuable in molecular breeding.

Chromosome DSSLs and TSSLs combined with corresponding SSSLs can be used to analyse the effect of epistasis interaction between QTLs, which is of importance for genetic analysis of complex traits and application of the target QTL in breeding. We developed three DSSLs (D1 to D3) and one TSSL (T1) using a MAS method, and observed that three SSSLs carried *qSSD5* or *qSSD8* or *qSSD10*, which positively affected seed-set density. The interaction between *qSSD5* and *qSSD8*, *qSSD5* and *qSSD10*, and *qSSD8* and *qSSD10* all produced negative epistatic effects in DSSLs. However, the sum of the additive and epistasis effects (genetic effect) on seed-set density of each double segment was greater than the value of the additive effect of the largest single QTL. Therefore, DSSLs showed a higher seed-set density compared with that of the single SSSLs. Thus, pyramiding of the QTLs that positively affected seed-set density resulted in a further increase in that trait. We also observed that TSSL T1, which carried *qSSD5*, *qSSD8*, and *qSSD10*, produced a higher seed-set density. These results were important for pyramiding favorable QTLs for yield-related traits.

### Molecular mechanism of rice panicle architecture and analysis of candidate genes for *qSSD5*, *qSSD8*, and *qSSD10*

Elucidation of the molecular mechanism underlying panicle architecture is important for breeding plants with high yield in rice. Considering genes related to development of rice panicles that have been cloned, the majority are associated with plant hormone signalling pathways or MAPK cascades. Auxin can affect plant architecture, and polar auxin transport plays a crucial role in plant growth and development (Zhang and Yuan 2014). Zhao et al. (2015) reported that *PAY1* improves plant architecture by affecting polar auxin transport activity and altering endogenous indole-3-acetic acid distribution, and ultimately affects rice panicle architecture and grain number. In our study, candidate gene 6 (auxin efflux carrier component) for *qSSD5* and candidate gene *OsSAUR32* for *qSSD8* also affected the distribution of auxin. The two genes displayed many SNP differences in sequence between Nipponbare and Z749. Furthermore, OsMPK proteins contains a highly conserved kinase domain. Mitogen-activated protein kinase (MAPK) cascades play a crucial role in plant growth and development as well as in biotic and abiotic stress responses (Reyna and Yang 2006). OsMKKK10–OsMKK4–OsMPK6 participates in rice panicle morphogenesis, and the GSN1–MAPK module mediates the trade-off between grain number and grain size by integrating localized cell differentiation and proliferation (Guo et al. 2018). In our research, we also observed many SNP differences in *OsMPK9* and *OsMPK17* between Nipponbare and Z749, the candidate genes for *qSSD5.* Although *OsMPK9* and *OsMPK17* have been cloned and *OsMPK17* is associated with pathogen infection (Hu et al. 2011; Reyna and Yang 2006), it is important to study further their effects on rice panicle development. Furthermore, we also checked the gene expression profile of all candidate genes for *qSSD5* and *qSSD8* using Rice eFP Browser website (http://bar.utoronto.ca/efprice/cgi-bin/efpWeb.cgi) and Rice expression database (http://expression.ic4r.org/global-search?gene). The results showed that eight candidate genes of *qSSD5* and two candidate genes of *qSSD8* all have higher expression level in rice panicles. Furthermore, we also performed qRT-PCR analysis in root, stem, leave, sheath, and panicle between Nipponbare and Z749 for these candidate genes. The results showed the consistence with the expression profiles in the public data, which displayed the highest or higher expression level in panicle. Among them, the expression level of candidate gene 3, 5 and 6 for *qSSD5* in panicle were the highest and the others were higher in panicle. However, only the expression level of candidate gene 6 for *qSSD5* in Z749 was higher significantly than in Nipponbare. Thus, the candidate gene 6 should be the best candidate gene of *qSSD5* for the difference in both DNA sequence and significant expression level between Nipponbare and Z749. The candidate gene 5 and 7 for *qSSD5*, and *OsSAUR32* for *qSSD8* differ only in DNA sequence and no difference in expression level between Nipponbare and Z749. Although four candidate genes for *qSSD5* and *qSSD8* have been identified by gene prediction, DNA sequencing, and gene expression level analysis, further functional complementarity verification together with candidate gene analysis of *qSDD10* are in progress. This should result in a more complete picture for panicle development for future research.

## Conclusions

Using the cultivar Nipponbare as the genetic background, we identified the rice CSSL Z749, which exhibits a dense and erect panicle phenotype. Z749 carried seven substitution segments derived from Xihui 18 with an average substitution length of 2.12 Mb. Fifteen QTLs were distributed on chromosomes 5, 7, 8, and 10 in Z749. The dense panicle of Z749 was caused predominantly by the reduction in panicle length and increases in the numbers of primary and secondary branches and the number of spikelets per panicle. The seed-set density of Z749 is controlled by one major QTL (*qSSD10*) and two minor QTLs (*qSSD5* and *qSSD8*). The QTLs *qSSD10, qSSD5*, and *qSSD8* were fine-mapped to intervals of 1.05 Mb, 1.46 Mb and 1.53 Mb, on chromosomes 10, 5, and 8, respectively. Sequencing analysis and qRT-PCR analysis revealed that two MAPK genes (*OsMPK9* and *OsMPK17*) and one gene that encodes an auxin efflux carrier component (candidate gene 6) might be candidate genes for *qSSD5*, and a small auxin-up RNA (*OsSAUR32*) might be the candidate gene for *qSSD8*. In particular, candidate gene 6 is the best candidate gene for *qSSD5*. Fourteen QTLs were verified by development of five SSSLs, and the repeatability of detection was 93.3 %. Epistatic effect analysis revealed that pyramiding of *qSSD5* and *qSSD8*, *qSSD5* and *qSSD10*, *qSSD8* and *qSSD10*, and *qSSD5*, q*SSD8*, and *qSSD10* all produced novel genotypes (D1, D2, D3, and T1) with a higher seed-set density.

## Materials and methods

### Experimental materials

The rice CSSL Z749 was developed using Nipponbare as the recipient parent and Xihui 18 as the donor parent. Xihui 18 is an excellent restorer line bred by Southwest University, Chongqing, China. A secondary F_2_ population raised from a cross between Nipponbare and Z749 was used for QTL mapping in 2018. On the basis of the QTL mapping results, nine F_2_ individuals were selected to develop SSSLs, DSSLs and TSSLs by MAS and grown as the lines Z775, Z776, Z777, Z778, Z779, Z780, Z781, Z782 and Z783 in 2019. In addition, three F_3_ segregated populations (Z784–Z786) for fine-mapping of *qSSD5*, *qSSD8* and *qSSD10* were developed from three recombinant plants for *qSSD5*, *qSSD8*, and *qSSD10* whose other genetic backgrounds was completely same with that of Nipponbare. Nine homozygous secondary substitution lines in F_4_ were planted in 2020 for validation of QTLs and epistasis effect analysis.

### Field planting

In 2017, Nipponbare and Z749 were crossed and the seeds were harvested at the experimental station of Southwest University in Chongqing, China. In autumn of the same year, the F_1_ hybrid seeds were sown at the Hainan experimental station and seeds from the F_1_ individuals were harvested. On 10 March 2018, seeds of Z749, Nipponbare, and the F_2_ population comprising 126 individuals were sown at the same experimental station in Chongqing, China. Thirty seedlings of each parent and all F_2_ seedlings were transplanted to the field on 20 April 2018, with 10 individuals planted per row. The spacing between rows and individual plants was 26.4 and 16.5 cm, respectively. Conventional management practices were applied. In 2019, five F_2_ individuals (Z775, Z776, Z777, Z778, and Z779) for the selection of SSSLs, three F_2_ individuals (Z780, Z781, and Z782) for the selection of DSSLs, one F_2_ individual (Z783) for the selection of TSSLs, together with individuals of Nipponbare were planted. For each material, 30 plants were transplanted. In addition, three segregation population (Z784–786) were planted for fine mapping of *qSS5*, *qSSD8*, and *qSSD10*, and all plants were transplanted at the same experimental station in Chongqing. In 2020, five SSSLs, three DSSLs, one TSSL, together with Nipponbare and Z749 were planted at the experimental base in Chongqing, again with 30 plants transplanted per line.

### Development of CSSL-Z749

First, 263 SSR markers polymorphic between Nipponbare and Xihui 18 were selected from 429 markers that covered the entire rice genome. Repeated backcrossing and selfing in combination with molecular MAS was used from the BC_2_F_1_ to BC_2_F_7_ generations derived from Nipponbare as the recipient parent and Xihui18 as the donor parent. In the BC_2_F_7_ population, a homozygous line with 13 substitution segments designated Z368, which produced dense and erect panicles, was selected. Nipponbare was crossed with Z368 and the progeny were selfed. Twenty individuals from each selfed generation were selected to develop CSSLs by MAS. Finally, CSSL-Z749 with dense and erect panicles, carrying seven substitution segments, was selected in the F_3_ population. The method of substitution segment identification followed that of Zhao et al. (2016a). The estimated length of the substitution segment was calculated in accordance with the method of Paterson et al. (1991). MapChart 2.2 software was used to draw the chromosome substitution segment map.

### Seed-set density related trait assessment

At maturity, 10 plants growing on the third to seventh hills of the central two rows of the Nipponbare and Z749 plots, and 126 individuals of the F_2_ populations, were harvested. The panicle length (PL), number of primary branches per panicle (NPB), number of secondary branches per panicle (NSB), number of spikelets per panicle (SPP), number of grains per panicle (GPP) and seed-set density (SSD) were measured. The specific measurement method used for each trait followed Ma et al. (2019). The seed-set density was determined as spikelets per 10 cm panicle length. A student’s *t*-test was used to analyse the significance of differences in these traits between Nipponbare and Z749. In addition, descriptive statistics, such as the average, standard deviation, skewness, and kurtosis in the F_2_ population, were calculated using Microsoft Excel 2010.

### QTL mapping

Total genomic DNA of Nipponbare, Xihui 18, Z749, and 126 plants from the F_2_ population was extracted using the cetyltrimethyl ammonium bromide method (McCouch et al. 1998). The procedures for PCR amplification, 10 % native polyacrylamide gel electrophoresis, and rapid silver staining were performed in accordance with the methods described by Zhao et al. (2016a). Nipponbare bands were scored as “−1”, Z749 bands were scored as “1”, heterozygous bands were scored as “0”, and the absence of marker bands was scored as “.”. The marker assignments for all markers on the substitution segments of Z749, together with the phenotypic values of each individual in the F_2_ population, were used for QTL mapping. Mapping of QTLs was performed using the restricted maximum likelihood (REML) method implemented in the HPMIXED procedure of SAS statistical software (SAS Institute Inc., Cary, NC, USA) with significance determined at α = 0.05.

### DNA sequence analysis of the cloned genes which might be allele of mapped QTL

Based on the QTLs or genes cloned previously, we speculated whether some of the genes are the causal genes of the QTLs that we mapped. We extracted the genetic information within the QTL mapping interval using Gramene (http://www.gramene.org/rice_mutant/) to predict the candidate gene and found three cloned genes, namely *OsFAD8* (*Os07g0693800*), *OsREL2* (*Os08g0162100*), and *OsPUP7* (*Os05g0556800*), in the corresponding region. We amplified the target gene fragments using genomic DNA of Z749 and Nipponbare as the template using primers for these five genes designed using Vector NTI 10. The PCR products were submitted to Tsingke Biological Technology Co., Ltd (Chongqing, China) for sequencing. The primers used for each gene are listed in Table S1.

### Additive and epistatic effect analysis of QTLs and multiple comparison for traits associated with seed-set density using SSSLs, DSSLs, and TSSLs

Given that only one substitution segment differed between each SSSL and its recipient parent, each of the SSSLs affecting a quantitative trait carried only a single QTL, and two overlapping SSSLs with a significant effect on the trait carried the same QTL. Identification of the QTL in the SSSL was performed via a Student’s *t*-test based on comparison of each SSSL with the recipient parent Nipponbare with significance determined at *α* = 0.05. Thus, the QTL controlling a trait that differed from Nipponbare existed on the SSSL when *P* < 0.05. The additive effect of the QTL was half the difference between each SSSL and Nipponbare (Zhao et al. 2007). Interaction of QTLs in each DSSL_ij_ was determined by comparing the difference between (Nipponbare + DSSL_ij_) and its corresponding SSSL pairs (SSSL_i_ + SSSL_j_) at *P* < 0.05. The epistatic effect in the DSSL_ij_ was estimated using half of the mean phenotypic values of [DSSL_ij_ + Nipponbare) − (SSSL_i_ + SSSL_i_)]. For the TSSL_ijk_, a student’s *t*-test was first used to detect significant differences between the traits (Nipponbare + Nipponbare + TSSL_ijk_) and (SSSL_i_ + SSSL_i_ + SSSL_k_), and when *P* < 0.05 it was considered that there was an epistatic effect between these QTLs. The epistatic effect in the TSSL_ijk_ was estimated using half of the mean phenotypic values of [(TSSL_ijk_ + Nipponbare + Nipponbare) − (SSSL_i_ + SSSL_j_ + SSSL_k_)] (Eshed and Zamir 1996; Zhang et al. 2020).

Finally, 5 traits associated with seed-set density of 10 plants of Nipponbare, S1-S5, D1-D3, and T1 were used to conduct Duncan’s multiple comparison using IBM SPSS Statistics 25.0.

### Fine mapping of *qSSD5, qSSD8*, and *qSSD10* and candidate gene analysis

Based on the results of the initial QTL mapping, three recombinant plants with only the “0” type band (heterozygous band type) at the *qSSD5*, *qSSD8*, and *qSSD10* loci and the other genetic backgrounds identical with Nipponbare were selected in the F_2_ generation. In the F_3_ population, 500 plants for *qSSD5*, 180 plants for *qSSD8*, and 400 plants for *qSSD10* were used for genetic analysis and linkage analysis for fine mapping. The primers for each gene are listed in Table S1. Within the interval for fine mapping of these QTLs, candidate genes were predicted according to annotations in the Gramene (http://www.gramene.org/), Rice Annotation Project (https://rapdb.dna.affrc.go.jp/), and China Rice Data Center (http://www.ricedata.cn/gene/index.htm) databases. Finally, primers for all candidate genes were designed to amplify the target gene fragments using genomic DNA of Z749 and Nipponbare as the template. The PCR products were submitted to Tsingke Biological Technology Co., Ltd (Chongqing, China) for sequencing.

### Total RNA extraction and qRT-PCR analysis

Total RNA was extracted from root, stem, leaf, sheath and panicle of Nipponbare and Z749 using the RNAprep Pure Plant RNA Purification Kit (Tiangen, Binjing, China). The first-strand complementary cDNA was synthesized from 2 µg of total RNA using oligo(dT)18 primers in 20 µL of reaction volume using the PrimeScript Reagent Kit with gDNA Eraser (Takara, Dalian, China). The qRT-PCR analysis was performed with a 7500 Real-Time PCR System (Applied Biosystems, Carlsbad, CA, USA) and a SYBR Premix Ex Taq II Kit (TaKaRa). Rice gene *Actin* (*LOC_Os03g50885*) was used as the internal control to normalize all data. Each set of experiments was repeated three times. All primer pairs used for qRT-PCR are listed in Table S1.

## Supplementary Information

**Additional file 1: Table S1.** Primers used in the study.

**Additional file 2: Figure S1.**. Expression level of *OsREL2* and *OsPUP7* between Nipponbare and Z749. A, B: Relative expression levels of *OsREL2 *and* OsPUP7* in root, stem, leaf, sheath and panicle between Nipponbare and Z749, respectively.

## Data Availability

The datasets supporting the conclusions of this article are included within the article.

## Abbreviations

QTL: Quantitative trait locus
SSSL: Single-segment substitution line
CSSLs: Chromosome segment substitution lines
DSSL: Double-segment substitution line
TSSL: Triple-segment substitution line
SSR: Simple sequence repeat
MAS: Marker-assisted selection
PL: Panicle length
NPB: Number of primary branches
NSB: Number of secondary branches
SPP: Spikelet number per panicle
SSD: Seed-set density
