# Supplementary material for: Identification, pyramid and candidate genes of QTLs for associated traits based on a dense erect panicle rice CSSL-Z749 and five SSSLs, three DSSLs and one TSSL
Source: Rice (N Y). 2021 Jun 16;14:55. doi: 10.1186/s12284-021-00496-7 (PMC8208356; doi:10.1186/s12284-021-00496-7)
Supplement: Supplementary file 1 — Additional file 1: Table S1. Primers used in the study. [file 12284_2021_496_MOESM1_ESM.docx]

**Supplemental Table S1. Primers used in the study.**

| Purpose | Primer name | Sequence | Remarks |
| --- | --- | --- | --- |
|  | SSR1-F | GATCACGTGCTTGTTCCGTAGC | SSR |
|  | SSR1-R | GGCTCTGCATCAATAAGCATAGG | SSR |
|  | RM3170-F | GCAGTGTCATTCTCATGAAACCTACC | SSR |
|  | RM3170-R | CAGACTCCAAAGCACCCATAACC | SSR |
|  | SSR2-F | TCATGCATGCTGATGACTGAAGG | SSR |
|  | SSR2-R | GGAAACGAACGGATGGACAGC | SSR |
|  | RM152-F | AAGGAGAAGTTCTTCGCCCAGTGC | SSR |
|  | RM152-R | GCCCATTAGTGACTGCTCCTAGTCG | SSR |
|  | RM22617-F | TAGACCGTTCTGACGTAATACCC | SSR |
|  | RM22617-R | CTAGACAACGTGGCTACCAAGC | SSR |
|  | RM1235-F  RM1235-R  RM1148-F  RM1148-R  RM5432-F  RM5432-R  RM5348-F  RM5348-R  RM8207-F  RM8207-R  RM3311-F  RM3311-R  RM4455-F  RM4455-R | GAGAAACACAATCAGTGACACC  CTGAAATTGCACTTCACTGG  CATGCTCGAATCAGTGAGTAGTGG  GCTTAGCTATGCAGGAGAGAGTGG  GCACAACAGTGACGGGCTGAGG  CAATGCATCGTTGTCTCAACAGTGG  TCACCGAATCCGATAGGAGTACC  CCTAAAGTGTATGGGCTGGAATGG  CGCCAACATCATCATCATCAACAGG  GTTCAACTTGGACGAGGATTTCATGG  CACTCGATGAGGCAATTTGAAGC  ATCATCCAAGTATCCCTGTCTCACG  TGCTCCGGAGATGTAGACTATGTCG  TTCGTGGGTGATGGATATCTGC | SSR  SSR  SSR  SSR  SSR  SSR  SSR  SSR  SSR  SSR  SSR  SSR  SSR  SSR |
|  |  |  |  |
|  |  |  |  |
| Mapping |  |  |  |
|  |  |  |  |
|  |  |  |  |
|  |  |  |  |
|  |  |  |  |
|  |  |  |  |
|  |  |  |  |
|  |  |  |  |
|  |  |  |  |
|  |  |  |  |
|  |  |  |  |
|  | OsFAD8-1-F | ACGTTAGCTTTAGGGTCATGT |  |
|  | OsFAD8-1-R | TCTACTCCTATCACCCTCCAT |  |
|  | OsFAD8-2-F | AACTTCCATAGCTTACCTGTGA |  |
|  | OsFAD8-2-R | ACATGACCCTAAAGCTAACGT |  |
|  | OsFAD8-3-F | ATGGAGGGTGATAGGAGTAGA |  |
|  | OsFAD8-3-R | CCTCTCAGCCACGCTACATTG |  |
|  | OsREL2-1-F | CTGGTTGCTGTCACTGGTAGCA |  |
|  | OsREL2-1-R | CCACCACTAGCTCAGAGCTAG |  |
|  | OsREL2-2-F | CCACCTGGTCTTGTTCAACCT |  |
| Sequencing | OsREL2-2-R | TGCTACCAGTGACAGCAACCAG |  |
|  | OsREL2-3-F | CTAGCTCTGAGCTAGTGGTGG |  |
|  | OsREL2-3-R | CAGCGTCCACGTCGAGTTGA |  |
|  | OsPUP7-F | TCCTAACCAGGTGTGCATGTGGT |  |
|  | OsPUP7-R | CCCATGCTATCGAAGCCTTCTCG |  |
|  | Candidate gene 1-1-F | ACCAACAACCACACACCTCCT |  |
|  | Candidate gene 1-1-R | CGATCAGAAATGTCCTGAACTG |  |
|  | Candidate gene 1-2-F | GTCAGCTTAACTCTGAAACATC |  |
|  | Candidate gene 1-2-R | AGGAGGTGTGTGGTTGTTGGT |  |
|  | Candidate gene 1-3-F | CAGTTCAGGACATTTCTGATCG |  |
|  | Candidate gene 1-3-R | TGCTGTGCCTTGCTATTACAC |  |
|  | Candidate gene 2-1-F | ATGGTCAAACTTTCAGGGTCTC |  |
|  | Candidate gene 2-1-R | CCAGGTTGGGATAGTCATCAGA |  |
|  | Candidate gene 2-2-F | CAAGCACCGTGCATGATGATGGA |  |
|  | Candidate gene 2-2-R | GAGACCCTGAAAGTTTGACCAT |  |
|  | Candidate gene 2-3-F | TCTGATGACTATCCCAACCTGG |  |
|  | Candidate gene 2-3-R | CCGTCATTCACACCTCGATGCA |  |
|  | Candidate gene 3-1-F | TGAATGTTGCTACTCCGAGCTAG |  |
|  | Candidate gene 3-1-R | ACTACTGCACAGCAGAGGCAT |  |
|  | Candidate gene 3-2-F | CGGGAGGCTGTACTCTACCAT |  |
|  | Candidate gene 3-2-R | CTAGCTCGGAGTAGCAACATTCA |  |
|  | Candidate gene 3-3-F | ATGCCTCTGCTGTGCAGTAGT |  |
|  | Candidate gene 3-3-R | CGGTAAGGGTCGGTGCTAAAC |  |
|  | Candidate gene 4-1-F | TATCGGTCGTAAGCGGATTTCG |  |
|  | Candidate gene 4-1-R | CTCCCAAAGCCACCATCATGTT |  |
|  | Candidate gene 4-2-F | CTGTGTTTCAGTTGCGTGTAT |  |
|  | Candidate gene 4-2-R | CGAAATCCGCTTACGACCGATA |  |
|  | Candidate gene 4-3-F | AACATGATGGTGGCTTTGGGAG |  |
|  | Candidate gene 4-3-R | TCTAATCAGGTGCGGACTCTGG |  |
|  | Candidate gene 5-1-F | GGTCTAGTAGTACGATCTCCTTCT |  |
|  | Candidate gene 5-1-R | ACAGGATCGGTGATTGTCCCAT |  |
|  | Candidate gene 5-2-F | CTACCTAGACAAGCGCACACA |  |
|  | Candidate gene 5-2-R | AGAAGGAGATCGTACTACTAGACC |  |
|  | Candidate gene 5-3-F | ATGGGACAATCACCGATCCTGT |  |
|  | Candidate gene 5-3-R | TGGAAAGGCGGAATGGTTATG |  |
|  | Candidate gene 6-1-F | CATCGCCAATCGCCTCCACTA |  |
|  | Candidate gene 6-1-R | CAGTCGGCAACAATCAGATGGT |  |
|  | Candidate gene 6-2-F | ACCAGCCACACTGACAATGCC |  |
|  | Candidate gene 6-2-R | CGAGGTATGTCCGTATGTGACACA |  |
|  | Candidate gene 6-3-F | ACCATCTGATTGTTGCCGACTG |  |
|  | Candidate gene 6-3-R | CAATGGAGACCAGTCACTTACG |  |
|  | Candidate gene 7-1-F | CACATGCCTGGTGAACTAATGT |  |
|  | Candidate gene 7-1-R | CGCGTAAACATCTCTGTTGTTC |  |
|  | Candidate gene 7-2-F | TGAGTTACGAACAGCTCATCC |  |
|  | Candidate gene 7-2-R | ACATTAGTTCACCAGGCATGTG |  |
|  | Candidate gene 7-3-F | GAACAACAGAGATGTTTACGCG |  |
|  | Candidate gene 7-3-R | AGAGCACAATACGGTGTGTACGG |  |
|  | Candidate gene 8-1-F | GTATCCAACTATTCTGTCGGCA |  |
|  | Candidate gene 8-1-R | CCGTATGTCATCTTTCACACAC |  |
|  | Candidate gene 8-2-F | CCTGGAGTATGTGCTGTAATGAT |  |
|  | Candidate gene 8-2-R | TGCCGACAGAATAGTTGGATAC |  |
|  | Candidate gene 8-3-F | GTGTGTGAAAGATGACATACGG |  |
|  | Candidate gene 8-3-R | CCAACTAGGTCGTCCCATTAG |  |
|  | LOC_Os08g02520-F | ATTGGTATATGAGGGATGTGC |  |
|  | LOC_Os08g02520-R | TATGCGATTGTTGCTCCATTC |  |
|  | LOC_Os08g02530-F | CCTCAGCATTGATCCTCTTCTG |  |
| LOC_Os08g02530-R | CAAACTGCTCTACCCAACTAGCT |  |  |
| OsFAD8-F | GTTATGTGGCTGGACTTCGT |  |  |
|  | OsFAD8-R | CATCACACCCTTTGCTGCTT |  |
|  | OsREL2-F | AATTGGAGTCTTCGAAGCAGAG |  |
|  | OsREL2-R | ACAACCATGGGGTAAACACTTC |  |
|  | OsPUP7-F | CCAAGGCCAAGAAGGTCG |  |
|  | OsPUP7-R | TCTCGACGTGTCCTCCGG |  |
|  | Candidate gene 1-F | GCTCGTCGGCAACTACTCCG |  |
|  | Candidate gene 1-R | CCCGATCCACCCGACCAT |  |
|  | Candidate gene 2-F | TCGACCGATCCGAGGGCCGT |  |
| qRT-PCR | Candidate gene 2-R | GTGGCACTGGCGGCCAATGT |  |
|  | Candidate gene 3-F | CAGGGTGATGGAGCTGATGG |  |
|  | Candidate gene 3-R | TGGTTCACCCGCAGCATCTC |  |
|  | Candidate gene 4-F | TCGTCGTGTACCTGCCGCA |  |
|  | Candidate gene 4-R | AGCTGCGCGTACACCTCGT |  |
|  | Candidate gene 5-F | GATGATCTCACACCAGAGCAT |  |
|  | Candidate gene 5-R | ATCGCAAACCTTCAGCTTG |  |
|  | Candidate gene 6-F | CTGCGGCTCATCCTCACTA |  |
|  | Candidate gene 6-R | CTAACCCTGCATCGGAGAGAAT |  |
|  | Candidate gene 7-F | ATGGAAGTGGCTCAGCAATTC |  |
|  | Candidate gene 7-R | AAGGTTCATCAGAGACAGGCC |  |
|  | Candidate gene 8-F | AAGCCCAAGTTCATTCCAT |  |
|  | Candidate gene 8-R | GACTTGCTTGCTACCATAAAC |  |
|  | LOC_Os08g02520-F | GGCGGAAGCGGTTCGTGAT |  |
|  | LOC_Os08g02520-R | CCACCGCAGCGGTGGCAGT |  |
|  | LOC_Os08g02530-F | GGGACACTTCGCGGTGTACG |  |
|  | LOC_Os08g02530-R | CAGTGGTCGAAGCCGTACTCCT |  |
|  | Actin-F | TGGCATCTCTCAGCACATTCC |  |
|  | Actin-R | TGCACAATGGATGGGTCAGA |  |
