# Supplementary material for: Identification, pyramid and candidate genes of QTLs for associated traits based on a dense erect panicle rice CSSL-Z749 and five SSSLs, three DSSLs and one TSSL
Source: Rice (N Y). 2021 Jun 16;14:55. doi: 10.1186/s12284-021-00496-7 (PMC8208356; doi:10.1186/s12284-021-00496-7)
Supplement: Supplementary file 2 — Additional file 2: Figure S1.. Expression level of OsREL2 and OsPUP7 between Nipponbare and Z749. A, B: Relative expression levels of OsREL2 and OsPUP7 in root, stem, leaf, sheath and panicle between Nipponbare and Z749, respectively. [file 12284_2021_496_MOESM2_ESM.pptx]

## Slide 1
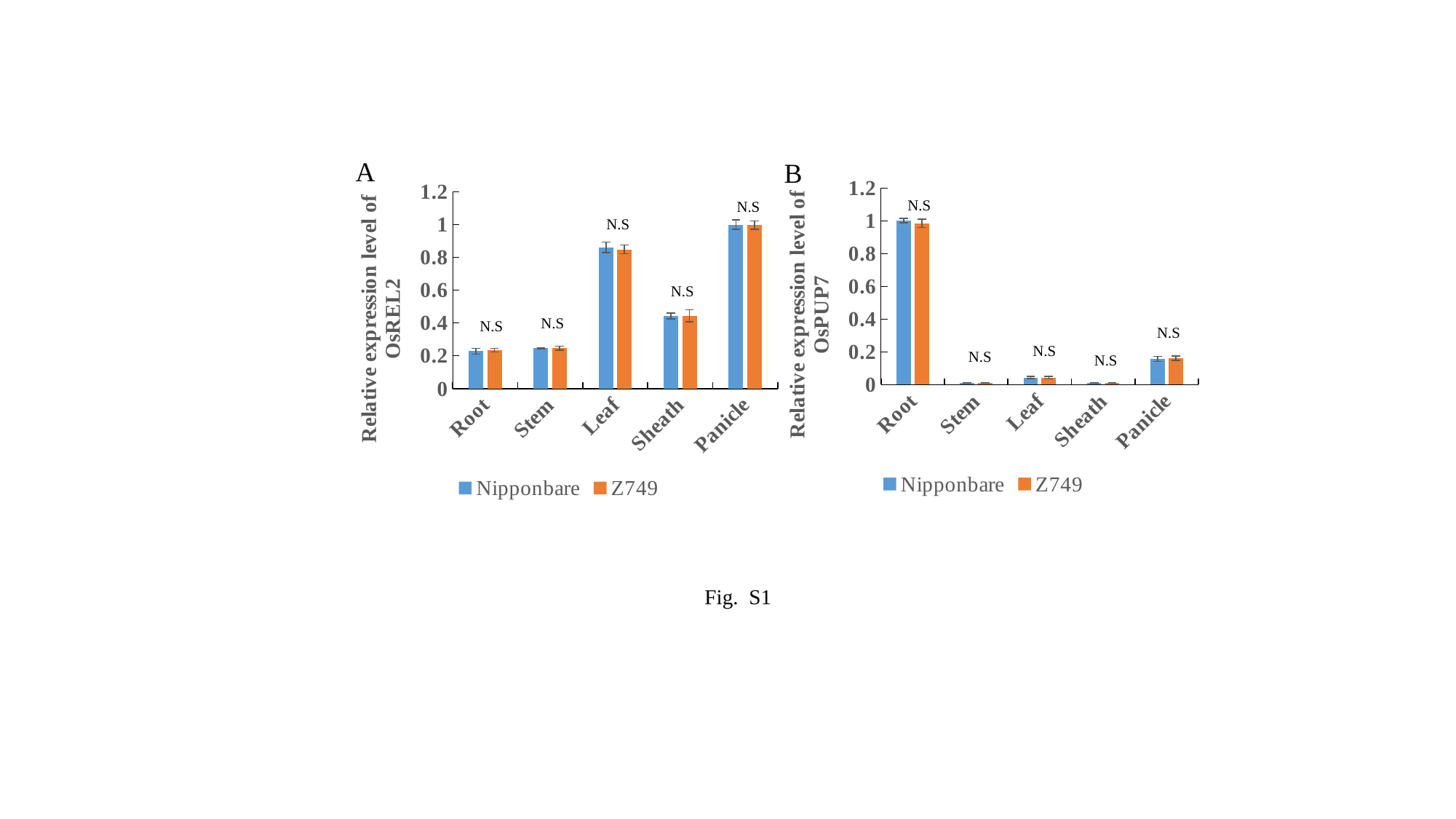

A
B
### Chart
| Category | Nipponbare | Z749 |
|---|---|---|
| Root | 1.0012158443966337 | 0.9849033501935204 |
| Stem | 0.00953808029876308 | 0.00921214809159466 |
| Leaf | 0.0436860439444137 | 0.04244958011078535 |
| Sheath | 0.01081395026318314 | 0.011681770502072475 |
| Panicle | 0.15679838357896778 | 0.16144571598821808 |N.S
N.S
N.S
N.S
N.S
### Chart
| Category | Nipponbare | Z749 |
|---|---|---|
| Root | 0.22803190303151796 | 0.2349688873051071 |
| Stem | 0.2472074530928167 | 0.24740180462108186 |
| Leaf | 0.8629152018309888 | 0.8501951760377313 |
| Sheath | 0.44364151425225523 | 0.4444879355023281 |
| Panicle | 1.0002739149235416 | 0.9986457169727533 |N.S
N.S
N.S
N.S
N.S
Fig. S1
